# Separation without Dissention: Observations Addressed to General Practitioners

**Published:** 1831

**Authors:** 


**Separation without Dissention: Observations addressed to General Practitioners. By Wm. Cooke, M.R.C.S. Octavo, pp. 32, 1831.**

Mr. Cooke has hit on a very ingenious method of issuing forth a second edition, without the trouble or expense of reprinting. The present pamphlet formed the concluding portion of a brochure published a few years ago; and, being now detached from its better half, is sent out into the world under the title at the head of this article. We do not say that there is any thing unfair in this; but we think the fact should be noticed, both in the title-page and the advertisements, in order that individuals may not have reason to complain that they have purchased the same work—or at least half of a work, twice over. At the same time, we are ready to admit that the observations contained in this pamphlet are well worthy of double, or even treble perusal. They are calculated, or at all events designed, to produce peace and good-will throughout all ranks of medical society.

From the following passage, we augur that the author is not an advocate for the recent proposal, to unite in one body the different grades of the profession.

"One chimerical suggestion (Mr. Green's)—of uniting the different orders of the profession into one class—with, indeed, some broad lines of division—has the sanction of a name that gives weight to the proposition;—and it is sent forth endorsed with a title well adapted to secure its acceptance. The author may have the credit of good intention, but as it bears on the General Practitioner (whose mental resources he does not duly appreciate) we are reminded of the trite anecdote of the man, who, being officious and troublesome to a commander and his crew during a tempest, was directed to hold a rope with all his might lest the vessel should be lost. The officers were relieved from the man's interference, and his self-importance was gratified, whilst in reality he had stood a cipher. With such differences of opinion as exist in the higher as well as lower departments, we are compelled to say with Horace,

——— ut nec pes, nec caput uni

Reddatur formæ.

It would greatly derogate from the interests of General Practitioners if they accepted of any concession (and some is undoubtedly due to them) that did not recognize an elevation of rank commensurate with their elevation of professional character. I am no advocate of a levelling system, and yet I think it both conducive to the advancement of medical philosophy, and congenial with the spirit of the times, that a door should be open to talent, though it may have germinated among the rubbish of pharmacy and obstetricy.

*It must, however, be remembered that as it relates to the public, and this is by far the most important consideration, legislation will effect little or nothing, except as it affords a security against incompetence. The practical application of knowledge and skill, so as to ensure confidence—to establish reputation—and to diffuse the inestimable blessings of the healing art, must rest with ourselves.*" PREFACE.

We apprehend that there is a great deal of truth in the concluding paragraph marked in Italics. If the rank of bishop or field-marshal were conferred on every individual in the medical profession, it would not advance their fame or fortune one iota. Nothing but cultivation of talent, exertion of industry, and the exercise of probity, will raise for a medical man either riches or reputation—of a lasting character; for we see too many instances of successful quackery, and scandalous traffic in human life for the sake of pelf. Laws and regulations are necessary for guarding against these unprincipled invaders of science—and also for securing to each individual a fair field for the exercise of his talents; but beyond this we are not very sanguine in our expectations of benefit from legislation. We conceive that the best plan, in this advanced æra, for equalizing *rights*, is to equalize *education*. Knowledge cannot be equalized—for that depends on exertions made long after the period of education is past. Whenever a uniformity of study and of examination is enjoined by law, for the medical profession, the summum bonum of legislation will be obtained.

